# Wearable Biosensors for Non-Invasive Sweat Diagnostics

**DOI:** 10.3390/bios11080245

**Published:** 2021-07-23

**Authors:** Jing Xu, Yunsheng Fang, Jun Chen

**Affiliations:** 1 School of Electrical & Electronic Engineering, North China Electric Power University, Beijing 102206, China; joycexu@ucla.edu; 2 Department of Bioengineering, University of California, Los Angeles, Los Angeles, CA 90095, USA; fys131415@g.ucla.edu

**Keywords:** point-of-care, biomonitoring, personalized healthcare, sweat, biosensors

## Abstract

Recent advances in microfluidics, microelectronics, and electrochemical sensing methods have steered the way for the development of novel and potential wearable biosensors for healthcare monitoring. Wearable bioelectronics has received tremendous attention worldwide due to its great a potential for predictive medical modeling and allowing for personalized point-of-care-testing (POCT). They possess many appealing characteristics, for example, lightweight, flexibility, good stretchability, conformability, and low cost. These characteristics make wearable bioelectronics a promising platform for personalized devices. In this paper, we review recent progress in flexible and wearable sensors for non-invasive biomonitoring using sweat as the bio-fluid. Real-time and molecular-level monitoring of personal health states can be achieved with sweat-based or perspiration-based wearable biosensors. The suitability of sweat and its potential in healthcare monitoring, sweat extraction, and the challenges encountered in sweat-based analysis are summarized. The paper also discusses challenges that still hinder the full-fledged development of sweat-based wearables and presents the areas of future research.

## 1. Introduction

The first wave of medical diagnostics saw the beginning of technological development in which people produced instruments that could measure almost any analyte of interest through the collection and transfer of samples to a separate lab. The second technological wave of POCT has even brought the lab into the hands of doctors, nurses, and even patients. More recently, a third technological wave is coming where patients can take the lab with them through wearable bio-marker monitoring [1,2,3]. Wearable technology can be an innovative solution to current medical problems with the ability to continuously monitor both physiological and biochemical markers and physical activities and behaviors [4,5,6,7,8]. Vital signs such as heart rate, body temperature, and blood pressure are measured [9,10]. Through the use of these signs, including electrocardiogram, electromyogram and biological fluid oxygen saturation and physical activity, the personal health condition can be characterized and monitored [11,12,13,14,15,16,17,18,19,20]. This paper reviews the method of chemical monitoring using sweat as the biological fluid [21,22,23]. Generally, personal items such as shoes, glasses, clothes, gloves, and watches can be used to deploy wearable devices [24,25,26,27]. As techniques move forward, emerging chemical wearable devices have evolved into devices that can be attached to the skin for further improving the accuracy of measurement. Furthermore, data collected by wearable devices and transmitted to a remote server through smartphones will be used to generate a personalized medical model through combination with analysis algorithms [28,29,30,31,32,33,34,35,36,37].

The need for wearable bioelectronics is rising daily and it has the potential to revolutionize the healthcare industry [38,39,40]. Conventional medicine is a reactive model, in which people wait for symptoms to manifest themselves in an individual before proceeding to diagnosis and eventual remedy. Due to this inherent nature of traditional medicine, it is quite often referred to as “Sick-care”. It is therefore desirable to move towards a preemptive model that can help people diagnose or take action to treat diseases at the earliest stages, well before the visible symptoms of the disease even become apparent. To move towards such a model, we need to understand how the body functions at a chemical or molecular level. This can be enabled through continuous non-invasive wearable biomonitoring [41,42,43]. The current system of a one-drug fits all approach is not accurate because people have varying body types and lifestyles. Personalized medicine can be modified according to each person’s characteristics that are acquired through the help of wearable biomonitoring [44,45,46,47,48,49,50,51,52,53]. As a result, our therapies will be more accurate since they rely on biomarkers rather than apparent symptoms [54,55,56,57]. Moreover, wearable bioelectronics can replace expensive and time-consuming lab tests with wearable diagnostic alternatives [58,59]. In traditional medical therapies or testing, many processes are involved, such as sample collection, preservation, and storage [60]. According to reports from the National Institutes of Health and the National Cancer Institute, adding preservatives or additives can potentially impact the protein makeup of plasma or any other bio-fluid involved in a particular test [61]. Storage temperature may also have a significant impact on sample quality. Moreover, temperature control issues may occur while transporting samples between facilities [62]. Altogether, even small errors can make a great impact on the test accuracy because all of them stack up and what reaches the lab is the summation of all of them. On top of these issues, these methods are expensive and time-consuming to complete the tests. Wearable testing measures over-ride this convoluted path by offering point-of-care-diagnostics (POCD) to the concerned individual at any time and any place while also saving time and cost.

**Figure 1:**
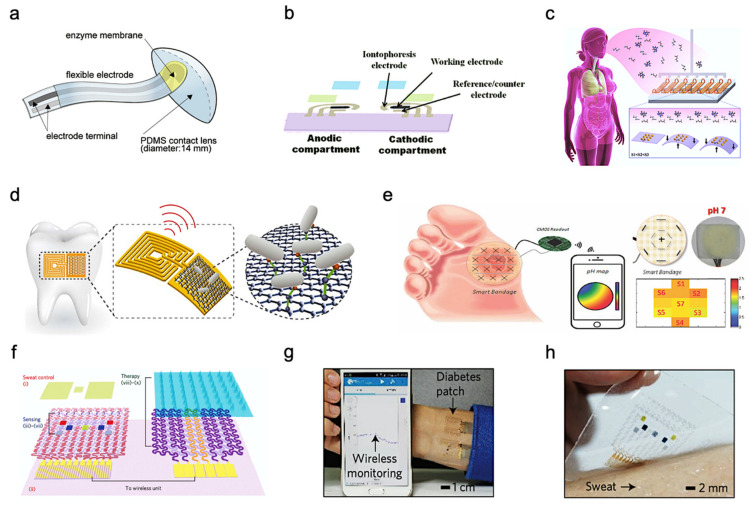
Wearable and flexible sensors for continuous biofluids analysis. (**a**) Soft PDMS contact lens with a glucose-sensing strip attached. Reproduced with permission from ref. [75], Copyright from 2020, Elsevier B.V. (**b**) All-printed tattoo-based ISF glucose sensor. Reproduced with permission from ref. [49], Copyright 2015, American Chemical Society. (**c**) Dynamic nanoparticle-based breath sensor. Reproduced with permission from ref. [78], Copyright from 2015, American Chemical Society. (**d**) Bacteria sensing on tooth enamel with graphene-based nanosensors. Reproduced with permission from ref. [79], Copyright from 2012, Nature Publishing Group. (**e**) Smart bandage for chemical sensing of wound pH using pH-sensitive threads. CMOS wireless readout and 2D mapping of pH levels were incorporated Reproduced with permission from ref. [74], Copyright 2017 IEEE. (**f**) Diabetes patch is composed of sweat-control (i, ii), sensing (iii–vii) and therapy (viii–x) components. (**g**) Integrated wearable diabetes monitoring and therapy system. (**h**) The electrochemical device on the human skin with perspiration. Reproduced with permission from ref. [80], Copyright from 2016, Nature Publishing Group.

Although there are many advantages of various wearable bioelectronics, non-invasive bioelectronics have their unique strengths and can also encourage users to take multiple readings in a day, as these tests are nondestructive and painless [63,64,65,66,67,68]. The large number of daily tests enabled by non-invasive wearable biomonitoring allows users to collect many data points across the population that can be used to gain a better understanding of diseases and will help us develop preemptive medical models to monitor healthcare before disease occurrence [69]. Great efforts have been made in wearable technologies as the acknowledgment of their uses on molecular biomonitoring has increased. These techniques are useful for sampling and analyzing the heavy metals, metabolites, toxic gases, and main electrolytes within body fluids [70,71]. Non-invasive detection of human body fluids generally includes the detection of tear, interstitial fluid (ISF), exhaustion breath, saliva, wound exudate, and sweat [72,73]. For example, in human tears, because biological fluids accumulate in the eyes, they contain certain salts, enzymes, proteins, and lipids. Consequently, eye conditions and diseases can be revealed through the analysis of the chemical composition of tears. Typically, increased levels of proline-rich proteins are considered biomarkers for the diagnosis of dry eye. In addition, tears have been used in the treatment of diabetes mellitus since the glucose concentration in tears is strongly correlated with the blood glucose concentration [74]. Figure 1a shows some of the tear-based flexible sensors developed using PDMS (Polydimethylsiloxane) or soft-lens [75]. Although blood biomarkers detection can provide more accurate reports, non-invasive detection is not possible for most of them. To circumvent this sampling problem, ISF monitoring is used to obtain information of chemical concentrations in blood alternatively. The composition of ISF is very similar to that of blood regarding the concentration of salt, protein, glucose, ethanol, and other small molecules [76,77]. A process called reverse iontophoresis is used on the skin to extract ISF. GlucoWatch, the most famous glucose sensing device approved by the United States Food and Drug Administration, uses this technology to carry ISF through the skin to an external sensor. It is an integrated wristwatch device with reverse iontophoresis and biosensor functions [74]. Figure 1b shows a wearable sensor for non-invasive monitoring of the ISF [49]. As for breathing, human breathing is a mixture of gases and vapors that we exhale through our nose or mouth. The composition of respiration is complex: it includes a mixture of nitrogen, oxygen, carbon dioxide, and water vapor [78]. In addition, respiration includes up to 500 different compounds, both endogenous and exogenous. The potential of respiratory measurement is huge because of its completely non-invasive and inherent safety. Figure 1c shows a wearable sensor for the non-invasive monitoring of human breath. Saliva is another attractive diagnostic bio-fluid that contains various disease signal biomarkers including hormones, enzymes, antibodies, antimicrobial agents, which can accurately reflect the status of humans [79]. These biochemical substances from the blood travel through spaces between cells and enter saliva. Thus, the main compounds found in the blood can also be detected in saliva, as shown in Figure 1d. Therefore, saliva is functionally analogous to the serum in that it involves changes in mood, hormones, nutrition, and metabolism. Salivary cortisol and salivary alpha-amylase, for example, are considered to be important biomarkers of physical and psychological stress. Proper monitoring of biofluids can also have benefits in the wound healing process. The formation or release of some compounds or exudates is crucial to wound healing. The pH value of the exudates, the concentration of uric acid and C-reactive protein can reflect wound healing progress and infection risk [74]. Figure 1e depicts research in a study in which wearable smart bandages were fabricated for wound monitoring. Among these bio-fluids, human sweat is crucial because it contains abundant physiological state information [80]. Figure 1f,g shows a sweat-based biosensor and its therapeutic system for wearable diabetes monitoring. The optical image of the device array on human skin with perspiration was shown in Figure 1h. There are a few advantages associated with sweat that make it a predominant candidate for most wearable biomonitoring research.

Since the distribution of sweat glands in the human body is rich (>100 glands/cm^2^) and the sweat contains abundant biochemical compounds, human sweat has become a promising bio-fluid for non-invasive biosensing [81]. Since nearly every portion of human skin has eccrine glands, sweat is readily available without the use of needles or other invasive devices. Iontophoresis sweat can be extracted from anywhere which is not possible in any other case of bio-fluids [69]. Moreover, analytes including ions [76], metabolites [82], acids [64,83], hormones [84,85,86,87], and small proteins [88,89] and peptides are partitioned into the sweat. Sweat also contains various electrolytes (such as potassium, sodium, chloride, and calcium), nitrogen-containing compounds (such as urea and amino acids), as well as metabolites such as glucose, lactic acid, and uric acid, along with xenobiotics such as drugs and ethanol [90,91,92]. This creates a huge opportunity for research and biomonitoring. Sweat chloride analysis has been used as the gold standard for the diagnosis of cystic fibrosis [93]. Sweat also has excellent sampling and detection efficiency without foreign contamination during testing and its composition does not affect the analytes to get degraded [81].

This review focuses on the recent progress made in the field of wearable and flexible bioelectronics for non-invasive health monitoring through in-situ sweat analysis. In the following sections, we will first discuss sweat partitioning and its relation to human health. Also, we will cover the most common sweat extraction techniques and fabrication methods for sweat-based bio-sensors. Mainly, we will review some of the recent researches and applications of continuous and non-invasive sweat-based biomarker monitoring, including health monitoring and disease detection, exercise monitoring, drug metabolism monitoring, and ethanol level measurement. Finally, we will go over the overall challenges and future scope of wearable sweat-based biosensors towards personalized health monitoring.

## 2. Sweat as a Bio-Fluid for Biomonitoring

Sweat can provide abundant biomarker measurements continuously and noninvasively of ions, drugs, metabolites and biomolecules, including potassium, sodium, calcium, chlorine, lactic acid, glucose, ammonia, ethanol, urea, cortisol, and various neuropeptides and cytokines. Table 1 also summarizes these key analytes in sweat and the detection methods. In addition to the abundant biochemical components in sweat, sweat glands are widely distributed across the human body. Consequently, sweat has become an ideal platform for noninvasive biosensing, which is feasible and safe as well. Sweat from the eccrine sweat gland can be noninvasive and easily obtained. This kind of sweat contains water and various electrolytes and can be directly excreted to the skin surface. Unusual health conditions (such as electrolyte imbalances and stress) and disease are usually reflected by changes in the concentration of existing sweat components or the emergence of new components in sweat. For example, the concentration of alcohol in sweat is highly correlated with the concentration of alcohol in the blood. The increase of urea concentration in sweat may be related to kidney failure. Moreover, because the concentration of chlorine in the sweat of patients who have cystic fibrosis (CF) is abnormally high, the analysis of chlorine in sweat has been regarded as a widely used method for the diagnosis of CF [74].

### 2.1. Sweat Partitioning

**Figure 2:**
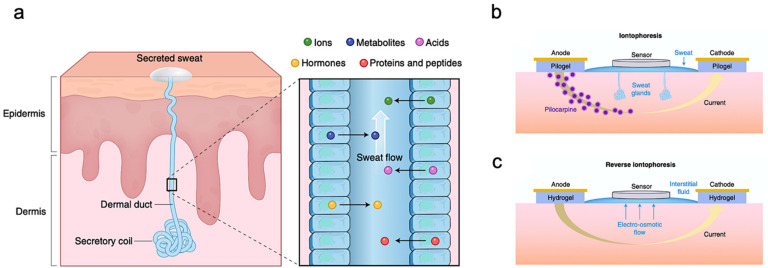
Sweat gland structure and biomarker partitioning. (**a**) Illustration of the sweat production process and metabolites that passed along with sweat. (**b**) Depiction of Iontophoresis to stimulate local sweat secretion at a selected site. (**c**) Reverse iontophoresis drives interstitial fluid through the epidermis to the skin surface. Reproduced with permission from ref. [69], Copyright from 2018, Nature Publishing Group.

Bio-marker partitioning in human sweat is important to study because the current gold standard for measuring biomarker concentration, for most biomarkers, is its concentration in blood. Therefore, correlating sweat biomarker concentration with the respective concentrations in the blood can help us draw meaningful inferences about the health of a person. In certain cases, independent concentrations in sweat can also have significant value. For example, chloride concentrations for patients with CF are very high in sweat independently and have been used in literature to diagnose CF [93]. The most easily obtained sweat comes from eccrine glands composed of coils and dermal ducts. These eccrine glands are the first place where sweat is produced, and the sweat is transported to the skin surface through dermal ducts [81], as shown in Figure 2a. During this process, analytes such as ions, metabolites, acids, hormones, small proteins, and peptides travel into human sweat. Sodium (Na^+^) and chloride (Cl^−^) ions have the highest concentration in sweat. Since they can stimulate hydration, they play a role in maintaining electrolyte balance in the human body. Ions such as Calcium (Ca^2+^) and Potassium (K^+^) also partition into human sweat from blood and are present in the mM (M for Molar) range [69]. K^+^ concentration can be used to predict muscle activity [104]. During exercises, electrical activity in the exercising muscles can cause an increase in K^+^ concentration. Weak acids or bases can also diffuse into the sweat gland and ionize because of the high pH of sweat. Other bio-marks, such as lactate and urea at mM levels, can come from human blood or be produced locally during the metabolic activity of the sweat gland. The lactate concentrations of human sweat are closely approximate to those of plasma [96]. Thus, the concentrations of lactate can indicate physical exertion and exercise intensity [105,106]. Sweat also contains larger molecules such as glucose, neuropeptides, and hormones, which are present in nM or pM traces. These important bio-marks in sweat carry valuable information about the human body. Take glucose as an example, monitoring the concentrations of glucose in sweat can provide continuous glycemic monitoring [102]. Apart from analytes that naturally be generated, drugs, heavy metals and alcohol can be discharged and detected in sweat as well when the human body tries to eject toxins. These biomarkers can be used for multi-purpose biomonitoring.

### 2.2. Sweat Extraction

Although it is a viable option to test sensors by sweat from exercise, realizing continuous sweat monitoring might not be practical. Under such circumstances, alternative methods of sweat extraction are needed. Iontophoresis is the most advanced non-invasive method to induce sweat excretion at a selected location such as the wrist [74]. It depends on local sweat stimulation through the application of topical current [69]. Figure 2b shows that the topical current is applied between pilogels, or hydrogels containing the sweat stimulant-charged drug, called pilocarpine. By applying a potential drop across the two sides of the test area, pilocarpine will be driven by a small current under the skin surface and trigger nearby glands to secrete sweat for wearable sensors to collect enough samples. This technology can gain unprecedented insight into the process of the sweat secretion, facilitating a wider range of sweat-based sensing applications such as health monitoring and POCD [93].

A process similar to iontophoresis is called reverse iontophoresis, which can be used to extract ISF by attaching two electrodes to the skin and applying a potential difference between them, as shown in Figure 2c. Ions, such as Na^+^, can be extracted outside of the skin. Since the net charge of the skin is negative, an electroosmotic flow will be generated to drive the interstitial fluid (ISF) to the skin surface through the epidermis without involving any drugs. The hydrogel isolates electrodes for applying current to the skin to prevent irritation. ISF-based wearable sensors can detect analytes, such as glucose, along with interstitial fluid transported to the skin surface by advection.

### 2.3. Sweat-Based Bio-Sensor Fabrication

There are certain fabrication methods usually involved in printing or making these sweat-based sensors into wearable devices. To make these flexible wearable sensors suitable for biomonitoring, several requirements should be satisfied. Firstly, the sensors must be highly sensitive, bio-compatible, and selective. Secondly, related circuitry should be in micro or nano size. Popular methods for this are photolithography and screen printing. Photolithography provides excellent resolution at the nanoscale by using an electron beam to write patterns. However, the cost of manufacturing is extremely high due to the equipment cost and clean room requirements. Low-cost screen printing can be used to produce large numbers of electrodes on various flexible substrates and can provide as high as one device per second throughput when roll to roll printing is used. However, the spatial resolution of the screen printing is not as good as that of lithography [106]. Other methods, such as epidermal elastomeric stamping, stamp transferring, and ink or aerosol jet printing, can also be used for fabrication. For example, stamp transfer and inkjet printing (combined with electroplating) showed great potential in high-resolution printing (down to about 2 µm). At the same time, it can provide manufacturing on non-planar substrates. Therefore, the appropriate selection and combination of these methods is ideal for the manufacture of sweat-based wearable bioelectronics.

## 3. Sweat-Based Wearable Bioelectronics

Wearable sweat-based sensors have been studied extensively recently for detecting analytes as they relate to human diseases and conditions. A few studies have also developed wearable devices with multi-analyte sensing and circuitry for in-situ analysis and calibration. In this section, we present some of these studies that illustrate the application directions of wearable technology, including health monitoring and disease detection, exercise monitoring, drug metabolism monitoring, and ethanol level measurement.

### 3.1. Health Monitoring and Disease Detection

**Figure 3:**
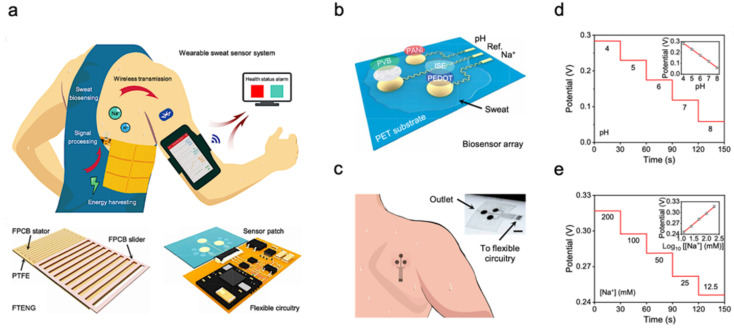
Sweat-based sensors for continuous health monitoring. (**a**) Describes the working of the device and shows the developed FTENG to power device. (**b**) Schematic of the sensor array. (**c**) Schematic of microfluidic sensor patch. (**d**,**e**) Open-circuit potential responses of the pH sensor in standard Mcllvaine’s buffer solutions (**d**) and a sodium ion sensor in NaCl solutions (**e**). Reproduced with permission from ref. [107], Copyright from 2020, the American Association for the Advancement of Science.

The molecular-level view of health is extremely valuable for health and disease monitoring applications. Sweat-based biosensors provide an effective way to achieve health monitoring and disease detection. For example, diabetes is directly related to the metabolite profile of glucose, which can be easily detected through sweat [44,98]. A sweat-based glucose monitoring system was developed using an electrochemical monolithic glucose sensor with pH and temperature correction functions [53]. The device can sense glucose levels in sweat in real time and even includes a drug that uses microneedles to regulate blood glucose. Although continuous blood glucose monitoring is becoming available, the potential of sweat sampling blood glucose monitors can not only reduce the size of current equipment but also achieve painless treatment of diabetes. Figure 3a shows a wearable molecular level health monitoring tool developed by researchers at Caltech which is self-powered by human motion [107]. The developed device was powered by a flexible triboelectric nanogenerator (TENG) [108,109,110,111,112,113,114]. It measures Na^+^ and pH of sweat using a potentiometric sensing technique, performs signal processing, and transmits this data to a mobile user interface using Bluetooth to track real-time personal health state. Figure 3b shows the schematic of a biosensor array containing both pH and sodium ion sensors patterned on a flexible plythylene terephthalate (PET) substrate [95]. The whole design is achieved in a wearable format by integrating it onto a flexible printed circuit board (PCB) that can be worn on the arm or the side of the human torso, as shown in Figure 3c. To realize the self-powering device, the authors developed a flexible TENG that can easily be integrated with the rest of the design. TENG bases on the principle of contact electrification and develops a potential difference between plates due to relative sliding between the copper and polytetrafluoroethylene plates. This helps the device power itself by harvesting energy from biomechanical energy induced by human motion. The developed TENG in this device manages to achieve a maximum power output of 0.94 mW for a 4.7 MΩ load, which is very suitable for wearable applications (Figure 3d,e). These results suggest that the device developed can be successfully used in molecular-level pH and Na^+^ monitoring [94,115]. Additionally, using a TENG to power the device itself enables it to achieve a very small form factor as it doesn’t use bulky batteries. It paves the way for developing innovative self-powering approaches for wearable devices used in human health monitoring.

**Figure 4:**
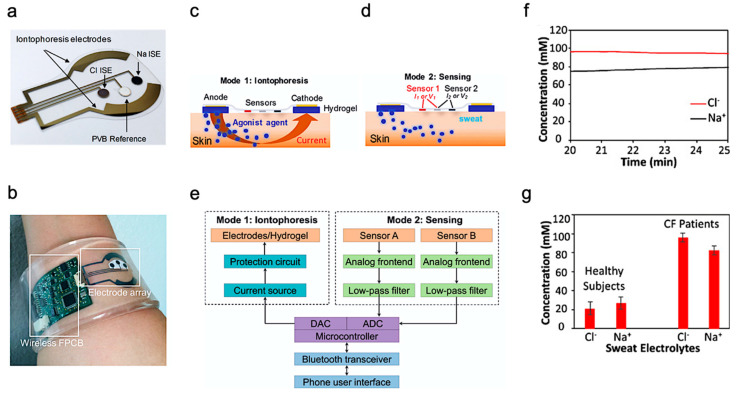
Overview of the developed CF monitoring device. (**a**) Electrodes used for iontophoresis and sensing. (**b**) Flexible wearable device on user’s wrist. (**c**,**d**) Description of the working modes of system (**c**) mode 1: Iontophoresis (**d**) mode 2: Sensing. (**e**) Block-level diagram of the developed system. (**f**) Real-time on-body measurement of sweat sodium ion and chloride ion levels of a CF patient after iontophoresis-based sweat stimulation. (**g**) Comparison of sweat electrolyte levels between six healthy subjects and three CF patients. Reproduced with permission from ref. [93], Copyright 2017, National Academy of Sciences USA.

Beyond health monitoring, sweat-based sensors can also provide low-cost disease detection and diagnosis. A wearable device was reported for the diagnosis of CF [93]. CF is a kind of hereditary disease which can cause severe damage to the human lungs, digestive system, and other organs. It makes secretions sticky by affecting cells that produce mucus, sweat and digestive juice. These viscous secretions no longer act as lubricants of the passage, but block the tube, catheter, and passageway, especially in the lungs and pancreas, causing serious damage to the human body. This leads to symptoms such as damaged airways, chronic infections, and in serious cases, even respiratory failure. According to the Cystic Fibrosis Foundation Patient Registry, more than 70,000 people worldwide are living with CF. Therefore, CF is a very serious disease that needs to be regularly monitored. The system-level implementation of the developed system is shown in Figure 4a,b. The working process of the device is described as follows—iontophoresis is used to induce sweat with various secretion profiles as depicted in Mode 1, which is then used for real-time sensing by the front-end electronics depicted in Mode 2 (Figure 4c,d). The processed signal is then transmitted to the communication module of the circuit, which sends the concentration data to the phone and displays it in a format that is easily understood by the user. The flow for signal processing is shown in Figure 4e. In this way, the system can measure the levels of sodium ion and chloride ion in the sweat of CF patients stimulated by iontophoresis in real-time (Figure 4f). Figure 4g shows the comparison results of sweat electrolyte levels achieved by the paper between six healthy subjects and three CF patients.

The results from the above study indicate its potential for use as a low-cost diagnosis of CF. Additionally, the demonstration of successfully integrating the design on a flexible substrate opens a plethora of possibilities for the development of wearable bioelectronics for disease diagnosis and monitoring.

### 3.2. Exercise Monitoring

**Figure 5:**
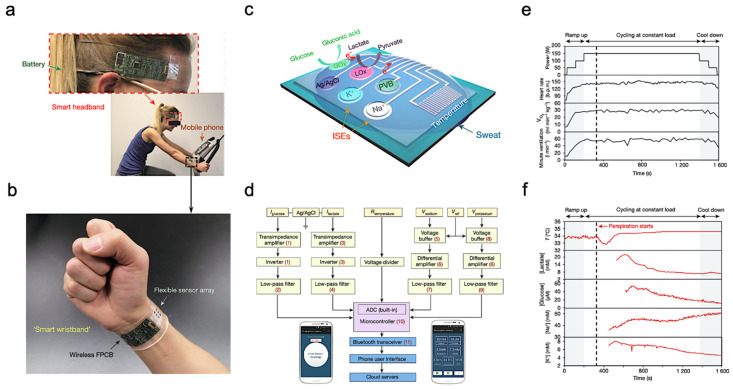
Sweat-based sensors for exercise monitoring. (**a**) Subject wearing forehead and wrist sensors undergoing stationary exercise. (**b**) Flexible integrated sensor array shown on a subject’s wrist as part of a wireless FPCB. (**c**) Schematic of the sensor array. (**d**) System-level block diagram illustrating the flow of information. (**e**) Real-time sweat analysis results of the FISA worn on a subject’s forehead. (**f**) Constant-load exercise at 150 W: power output, heart rate (in beats per minute, b.p.m.), oxygen consumption and pulmonary minute ventilation, as measured by external monitoring systems. Reproduced with permission from ref. [44], Copyright from 2016, Nature Publishing Group.

The most common application of wearable sweat biosensors is exercise monitoring. In the study of Gao et al., a wearable sensor array was introduced where several analytes could be monitored at the same time. The sensor used a flexible integrated sensing array (FISA) and the signal was processed by a flexible PCB (FPCB) [44]. The integrated FISA and FPCB are displayed and function on a subject’s head and wrist, as shown in Figure 5a,b. These sensors need to bear the stresses of everyday wearing and physical exercise. Upon bending FPCB at 1.5 cm and 3 cm bending radii, minimal change in output was observed in FISA response.

Figure 5c illustrates the structural design and working mechanism of the sensor. The working principle of Amperometric glucose and lactate sensors is immobilizing glucose oxidase and lactate oxidase inside a permeable membrane. Both the shared reference electrode and the counter electrode of the two sensors use Ag/AgCl as the electrode. Current signals proportional to the corresponding metabolites will be generated through these enzymatic sensors automatically and transported between the working electrode and the Ag/AgCl electrode. Ion-selective electrodes (ISEs) were used to measure the Na^+^ and K^+^ levels. The ion-selective electrode is a type of potentiometric device that combines with a reference electrode coated with polyvinyl butyral (PVB) to stabilize the potential in solutions with different ionic strengths. In addition, a resistance-based temperature sensor is achieved through the fabrication of Cr/Au microwires.

Figure 5d shows the measured potentiometric data is transmitted through an amplifier, an inverter, an analog-to-digital converter, and a Bluetooth module to be displayed on a cellphone. The sensor was tested on a subject undergoing a stationary cycling exercise as shown in Figure 5a. Specifically, the exercise program includes 3 min of accelerated cycling, 20 min of fixed 150 W cycling, and 3 min of cooling down. During exercise, heart rate, oxygen consumption, and pulmonary ventilation were measured. It showed that the values of the measured data increased in proportion with the increase of output power. Figure 5e illustrates the real-time measurement of sweat on a subject’s forehead using FISA. It shows that the skin temperature increases at about 400 s and stays almost unchanged after that with continuous perspiration. Meanwhile, lactic acid and glucose concentrations in sweat gradually decrease, as shown in Figure 5f. This decrease in lactic acid and glucose can be attributed to the dilution effect caused by the increased per-spiration rate. 

**Figure 6:**
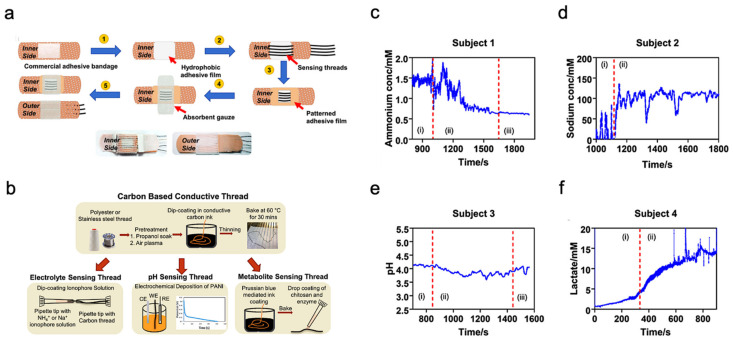
Sweat-based multimodal sensor patch for exercise monitoring. (**a**) Step-by-step process of fabrication and complete sensor patch prototype. (**b**) Fabrication of specialized sensing threads. (**c**–**f**) Measurement results of different biomarkers. Reproduced with permission from ref. [116], Copyright from 2020, Nature Publishing Group.

Unlike typical use of polymeric substrates, a textile-based platform can increase overall permeability to the affected skin area, which cultivates natural sweating and evaporative cooling [116]. This sensor is constructed from different layers of materials, as shown in Figure 6a. A commercial adhesive bandage is used as the base for the sensor, then a hydrophobic adhesive film is placed above to retain sweat samples within the patch. Additionally, specialized sensing threads are implemented in a parallel pattern to the previous layer. An absorbent gauze is applied to further enhance the collection of the sweat sample. Eventually, an adhesive film is placed on top to keep the entire patch intact.

The thread bundle incorporated within the sensor patch contains two specialized thread types for analyzing the biomarkers within the sweat sample. These specialized threads are commonly available threads that have undergone additional treatments and processes; the specific fabrication details of the threads are shown in Figure 6b. The first thread type in discussion is a carbon-based conductive thread that can sense the electrolyte, pH, and metabolite levels within the sweat sample. These measurements directly relate to biomarkers such as sodium, lactate, and ammonium which can represent real-time physiological status of an individual undergoing strenuous physical activity. The second thread type is a silver-silver chloride thread that acts as a solid-state reference for comparison during analysis. The four graphs shown in Figure 6c–f displayed the sensor patch performance when applied onto a participant’s arm while exercising on a stationary bike. Each graph is sectioned by Roman numerals with ”1” as equilibrium state, ”2” as real-time measurement, and ”3” as cool-down period. The results demonstrate the consistency and stability of the sensor patch in collecting data from the biomarkers within the sweat sample.

### 3.3. Drug Metabolism Monitoring

**Figure 7:**
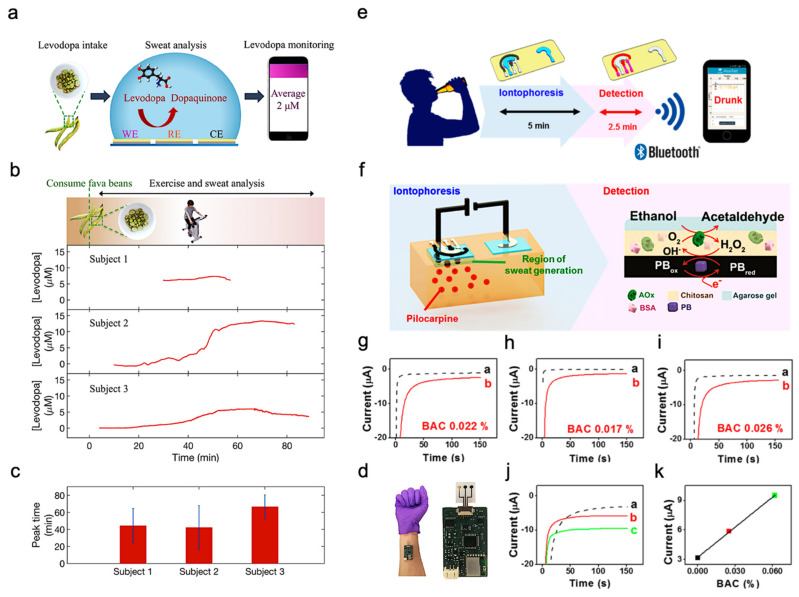
Drug sensing mechanism and ethanol levels measurement. (**a**) Interaction of the sensor with sweat. (**b**) Cycling and sweat analysis. Examples of sweat levodopa concentrations for three different subjects after they consume 450 g of fava beans. (**c**) Averaged time of peak levodopa concentration for three different subjects across multiple exercise trials. (**d**) Optical image of the S-band worn on a subject’s wrist. Reproduced with permission from ref. [99], Copyright from 2019, American Chemical Society. (**e**) Sweat ethanol sensor used to send alerts to a smart device. (**f**) Schematic diagram of constituents in the iontophoretic system (left) and of the reagent layer and processes involved in the amperometric sensing of ethanol on the working electrode (right). (**g**–**i**) Experiments with consumption of 12 oz of beer measured from three different human subjects before (plot “a”) and after drinking alcohol beverage (plot “b”). (**j**) Chronoamperograms obtained from a: BAC 0%, b: BAC 0.025% and c: BAC 0.062%. (**k**) Correlation between current response and the BAC level. Reproduced with permission from ref. [101], Copyright from 2016, American Chemical Society.

In addition to exercise intensity monitoring, wearable sweat-based sensors for bio-marks monitoring can also be applied as drug metabolism monitoring sensors. For example, Levodopa is used to treat patients suffering from Parkinson’s disease. Various factors can affect an individual’s response to levodopa and therefore it is important to monitor the concentration in blood. Since blood-based monitoring is usually invasive, sweat-based levodopa sensing was investigated using a sensor packaged into a sweatband (S-band) [99]. The sensor has a standard three-electrode (working electrode, reference electrode and counter electrode) design and is fabricated on a PET substrate (Figure 7a). Experiments were performed on healthy subjects after consuming fava beans as they happen to contain levodopa. In this way, the function of the S-band can be extensively tested on non-vulnerable subjects, and sweat was elicited from the subjects using iontophoresis. The concentration of levodopa in sweat was continuously monitored after consumption of fava beans and followed by iontophoresis. The S-band can detect the level of levodopa in sweat continuously, which is similar to the level in blood.

Sweating caused by iontophoresis lasts only for a very limited time period, while that caused by exercise generally lasts longer. This is helpful in the experimentation and validation of these sensors. Figure 7b shows the response observed from three subjects exercising on a stationary ergometer. In each trial, a subject would consume 450 g of fava beans and undergo multiple exercise trials. Cumulative results are shown in Figure 7c, depicting the average time of peak concentration. By analyzing the sweat induced by physical activities and iontophoresis, it is possible to optimize the dose of drugs by monitoring drug metabolism. Figure 7d shows the sensor apparatus mounted on a subject’s wrist. The future development directions include the study of pharmacodynamics between drugs, prolonging the duration of iontophoresis sweating, and improving the lifetime of the electrode. As a result, S-band can be used to study the inherent complex drug profiles, optimize drug dosages for people with Parkinson’s disease, and be incorporated into drug delivery systems.

### 3.4. Ethanol Levels Measurement

Sweat ethanol levels are an indicator of blood alcohol concentration. Ethanol levels were measured using a sweat-based sensor in tattoo form [101]. Figure 7e presents the illustration of a system that uses a sweat ethanol sensor to send alerts to a smart device. A tattoo-like patch based on an enzyme amperometric sweat ethanol sensor was developed with a pilocarpine iontophoresis drug delivery system and connected to intelligent devices through a Bluetooth module. The external current of iontophoresis (0.6 mA) was optimized between the effective delivery of drugs and the comfort of subjects because high current can easily cause skin irritation. The device is more reliable than the commonly used breath meter because it avoids potential errors induced by environmental factors like water vapor, or consumer products like mouthwash. Compared to other transcutaneous devices, it is a faster blood alcohol concentration measurement method because it only takes about 10 min compared with 0.5–2 h on traditional devices. Therefore, it is suggested that ethanol sensors can be used to detect illegal levels of alcohol consumption in car drivers.

This sensor system uses iontophoresis technology with constant current to induce sweat by delivery of the drug pilocarpine through the skin to perform sweat ethanol sensing. The sensor uses an alcohol oxidase enzyme electrode and a printed Prussian blue (PB) electrode transducer. All the electrodes on the wearable temporary tattoo paper are produced by screen printing for mass production and can be removed from the skin easily. Figure 7f illustrates these processes starting with iontophoresis followed by amperometric detection. Experiments were performed on three different human subjects using this sensor to validate the response of the system. Figure 7g–i shows plots that illustrate the responses of the subjects upon consumption of equal amounts of alcohol. Curve a is the amperometric response before drinking, and curve b is after drinking an alcoholic beverage. The blood alcohol concentrations of these subjects are different due to varying metabolism rates. However, after consuming alcohol, the current change from the sensor is quite apparent. Three control experiments were conducted to make sure there are no false positives. Figure 7g shows that zero blood alcohol content (BAC) results in no change in sensor response. And the sensor response after a certain amount of time had elapsed without alcohol consumption. This proves the sensor current response shown was caused by alcohol consumption. Figure 7h shows the response if no enzyme is immobilized in the sensor, showing that it is highly specific to sweat ethanol levels. Figure 7i shows the sensor response with and without iontophoresis, showing that the method of sweat extraction does not affect the sensor response. Thus, the sensor is highly specific to sweat ethanol levels and does not produce a response with 0 BAC. Figure 7j showed the sensor response at different BAC, with (a) being at 0%, (b) at 0.025%, and (c) at 0.062%. Figure 7k showed current changes at different BAC.

Additional control experiments were performed to validate the sensor. It has been shown to provide reliable information in real-world settings, which can provide a highly useful instrument to monitor alcohol for road safety. A more interesting implementation could be to fit these devices in such a way that the measured BAC determines whether a vehicle can be started or not. Future systems would involve calibrating the device, ensuring data security, and safeguarding privacy.

### 3.5. Biomolecules Monitoring

**Figure 8:**
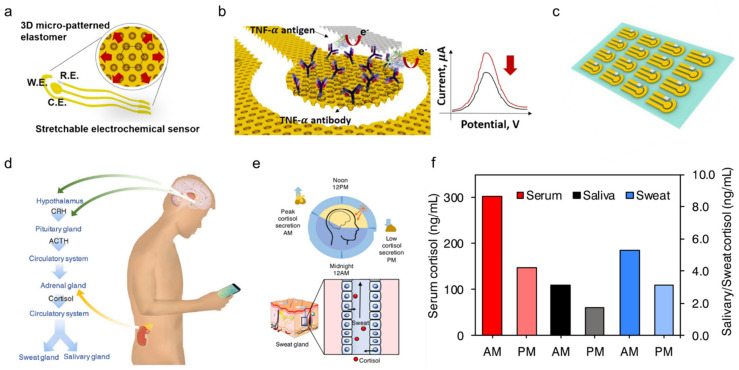
Sweat-based sensors for biomolecules monitoring. (**a**) Schematic showing a stretchable chemical immunosensor for TNF-α cytokine sensing. (**b**) Schematic illustration of the TNF-α cytokine proteins immobilized on the working electrode for electrochemical sensing. (**c**) Image of fabricated device arrays. Reproduced with permission from ref. [103], Copyright from 2019, Elsevier. (**d**) Schematic illustration of cortisol in sweat and saliva. CRH, corticotropin-releasing hormone; ACTH, adrenocorticotropic hormone. (**e**) Schematic showing the light and dark cycle for regulating circadian rhythm and controlling of cortisol transport to sweat. (**f**) Cortisol levels in serum, saliva, and sweat at different times from a healthy subject. Reproduced with permission from ref. [27], Copyright from 2020, Elsevier.

Compared to the three biomarkers mentioned, biomolecules including proteins, cytokines, nucleic acids, or neuropeptides are also important indicators reflecting a subject’s health or infection status. Although they are always present at relatively low concentrations in physiological fluids, they are of great interest for monitoring the chronic wound healing process as well as future diagnosis or management of diseases such as wound healing, Parkinson’s disease, and depression. As an example, a stretchable electrochemical immunosensor was used to detect the protein of the TNF-*α* antibody to monitor wound healing (Figure 8a) [103]. TNF-*α* was immobilized on the working electrode and detected using a voltammetric technique of differential pulse voltammetry method. Without the addition of TNF-*α*, the Faraday current was recorded at the redox potential that came from the redox of ferricyanide (Figure 8b). When TNF-*α* is added, a barrier layer is formed on the surface of the working electrode to inhibit electron transfer to reduce the recorded current. The immunosensor shows decent sensing performance both in buffer solutions with clinical concentration ranges (0.1 pM–0.1 μM) and human serum. Additionally, it can adapt to strains up to 30% due to 3D micro-patterned elastomers as a potential for body-attachable immunosensing (Figure 8c).

Besides, timely and accurate detection of stress is essential for monitoring and managing mental health. Considering that the current questionnaire and other methods are very subjective, a wearable chemical sensor was proposed with a highly sensitive, selective, and miniaturized mHealth device based on a laser flexible graphene sensor to non-invasively monitor the level of the stress hormone of cortisol (Figure 8d). It shows a strong correlation between sweat and circulating cortisol and demonstrates that changes in sweat cortisol respond quickly to acute stress stimuli. In addition, it showed a diurnal cycle and sweat cortisol pressure response curve, revealing the potential for dynamic pressure monitoring achieved by the mHealth sensor system (Figure 8e). Trends in ante meridiem/post meridiem (AM/PM) cortisol variability modulated by circadian rhythm are observed from a subject with the ratios ranging from 1.35 to 2.00 (Figure 8f). This platform provides a rapid, reliable, and decentralized health care vigilance at the metabolic level, thereby guiding an accurate snapshot of our physical, mental, and behavioral changes. Other sweat based wearable bioelectronics are also developed to detect pathogenic contamination [117] and antimicrobial peptides [79], and are compatible with the customized display interface for more convenient monitoring of health performance.

## 4. Conclusions and Future Scope

In the new age of wearable bioelectronics, sweat-based wearable bioelectronics has emerged as a leading technology that can measure biomarkers from a source that is boundless and easily extractable. The acquisition of biomarkers is generally noninvasive, will not degrade analytes, and has high sampling and detection efficiency. Recent advances in sweat-based wearables have also demonstrated a strong indication that this technology can be utilized in: health monitoring and disease detection, exercise intensity monitoring, drug metabolism monitoring, and ethanol level measurement. To further promote the practicality of sweat-based wearable bioelectronics, the following challenges need further attention:

(1) Improvement of biomarkers availability in sweat. Although sweat contains many biomarkers, the concentration of these biomarkers varies widely. In general, the concentration of biomarkers in sweat is significantly lower than that in other body fluid samples with a similar volume. Compared with human plasma, the proportion of sodium, potassium, lactic acid, and glucose in sweat is small. The main reason behind this huge difference is the filtration of extracellular matrix tight junctions, which limits the size of molecules that can pass through the skin. Therefore, improving the availability of biomarkers in human sweat is one of the key problems to be solved in sweat-based wearable bio-electronic devices.
(2) Sustained sample source and stable quantity. It is well known that the amount of skin perspiration varies from individual to environment. Therefore, the ideal wearable sensor must be able to accommodate the variations of different individuals and provide accurate sweat monitoring. Continuous monitoring of sweating is difficult to achieve because a person cannot sweat for a long time without external stimulation. This special problem greatly reduces the efficiency of an independent sweat-based wearable device for continuous monitoring throughout the day. Although we can use some methods to induce perspiration for providing sample sources for wearable sweat sensors, the differences in composition between heat-induced perspiration and chemical-induced perspiration are still questionable. Currently, it seems very convenient and time-saving to use chemicals such as pilocarpine to artificially produce sweat, and it can provide sweat samples continuously and indefinitely. However, it is not yet clear whether this sweating stimulation will affect the individual’s common sweating function and induce health concerns of long-term use in the human body.
(3) Improvement in sample quality. The quality of sweat samples is also susceptible to various external factors which may lead to inaccurate measurement data. For example, sweat produced during strenuous physical activity is usually used to cool the body temperature. Thus, the sweat tends to evaporate on the surface of the skin to carry away heat for cooling purposes. Consequently, the concentration of biomarkers in the initial sweat sample changes during evaporation. In addition, the sweat excreted by the human body is easily contaminated by pollutants on the surface of the skin. In research settings, a protective conductive layer is added between the sensor and the skin area where the sweat is artificially induced to prevent the generation of pollutants. However, the dead volume between surfaces may cause a delay in the time from perspiration to sensing, which reduces the accuracy and increases the latency of the collected data. To improve the measurement accuracy of the sensor, more attention and effort should be paid to this problem.

Although with numerous challenges yet to be overcome, sweat-based sensors for biomonitoring are a nascent and promising field of technology. Several technological challenges will need to be resolved, as listed above, before a commercial implementation of wearable sweat-based devices can be developed. Some future directions for further research and development are also discussed here:

(1) Exploring efficient power supply methods. The great progress of wearable biosensor technology and the growing demand for multi-task processing on wearable platforms to promote the development of advanced power supplies. To realize non-invasive wearable bioelectronics, the power supply should be efficient and sustainable and have good flexibility to meet skin contour and mechanical stress. Although great efforts have been made in noninvasive flexible fuel cells and biofuel cells, the current technology is far from the requirements to provide stable and reliable power support for most of the existing wearable bioelectronics. Besides further boosting the power output from the flexible fuel cells, another possible solution is to manufacture microsensors that consume less power. Additionally, harnessing energy from multiple sources, such as biomechanical energy and solar energy, could also be a promising solution.
(2) Developing suitable data processing and system integration methods. To obtain informative results gathered from wearable bioelectronics, it is necessary to perform appropriate post-processing on the electrical signal of the sensor, including amplification, filtering, and analog-digital conversion. Then, the processed signal is transmitted to the upper computer for analysis and display. Therefore, in this process, the electrical signal gathered by the sensor needs to be sampled by the processor and converted into a recordable value. The sampled raw data may suffer from inherent or environmental noise. Appropriate signal processing methods can reduce the influence of these noises, which is conducive to extracting useful signals from the sensor. The processed data is then transmitted to an external platform, such as a computer or mobile phone, for displaying and analysis. Here, the major role of data processing is to reduce noise and provide a user-friendly display of the recorded data. For applications requiring big data storage space and complex calculations, the data needs to be preprocessed before transmission. At present, the most popular technologies used for real-time data streaming and analysis in wearable sensing devices are low-energy Bluetooth and near field communication (NFC). However, both technologies have obvious transmission drawbacks. For example, NFC needs to be close to the receiver electronics for functioning. A transmission system that achieves the ideal connection has yet to be developed.
(3) Reducing the delay of data collection and analysis. Since it is a complex process to analyze and process the data collected by the sweat sensor, it generally takes a long time to complete. However, during this process, the evaporation of sweat on the skin surface will cause changes in the concentration of biomarkers in the sweat sample, which will pose a major obstacle. Reducing the delay between sweat collection and analysis is an important research area, which may be solved by developing low-power and high-performance microprocessors. Moreover, advanced big-data processing methods based on machine learning or deep learning algorithms can be further integrated into the system to realize the rapid and accurate extraction of the collected data.

## Figures and Tables

**Table 1:** Key analytes in sweat and the related detection methods.

Target Analyte		Concentration in Sweat	Recognition Element	Sensing Modality	Ref.
Ions	Na^+^	10–100 mM	Na+	Potentiometry	[44,45,93]
	Cl^−^	10–100 mM	Ag/AgCl	Potentiometry	[93,94]
	K^+^	1–18.5 mM	K+	Potentiometry	[44,94]
	Ca^2+^	0.41–12.4 mM	Ca2+	Potentiometry	[95]
	pH	3–8	Polyaniline	Potentiometry	[96]
	NH^4+^	0.1–1 mM	NH4+	Potentiometry	[97]
	Zn^2+^	100–1560 μg L^−1^	Bi	Square wave stripping voltammetry	[84,98]
	Cd^2+^	<100 μg L^−1^	Bi	Square wave stripping voltammetry	[98]
	Pb^2+^	<100 μg L^−1^	Bi, Au	Square wave stripping voltammetry	[98]
	Cu^2+^	100–1000 μg L^−1^	Au	Square wave stripping voltammetry	[98]
	Hg^+^	<100 μg L^−1^	Au	Square wave stripping voltammetry	[98]
Drugs	Levodopa	<10 μM	Au	Chronoamperometry	[99]
	Caffeine	<40 μM	Carbon	Chronoamperometry	[100]
	Alcohol	2.5–22.5 mM	Carbon	Chronoamperometry	[81,101]
Metabolites	Glucose	10–200 μM	Glucose oxidase	Chronoamperometry	[44,93,102]
	Lactate	5–20 mM	Lactate oxidase	Chronoamperometry	[96]
	Uric acid	2–10 mM	Carbon	Cyclic voltammetry	[83]
	Cortisol	8–140 μg L^−1^	ZnO, MoS2	Electrochemical impedance spectroscopy	[85,86]
	Ascorbic acid	10–50 μM	Carbon	Chronoamperometry	[83,87]
Biomolecules	Peptides	0.1 pM–0.1 μM	Au	Chronoamperometry	[103]
	Antimicrobial peptides	-	Carbon	Resistance	[79]

## Data Availability

Data of our study are available upon request.
